# A Diagnostic Dilemma of Antiglutamic Acid Decarboxylase 65 (Anti-GAD 65) and Mycoplasma Pneumoniae Antibodies in a Girl Presenting with Acute-Onset Obsessive-Compulsive Disorder

**DOI:** 10.1155/2021/6672028

**Published:** 2021-03-19

**Authors:** Cecilia G. Freeman, Antanoid J. Langeveldt, Robyn R. Miller

**Affiliations:** ^1^Sidney Kimmel Medical College, Thomas Jefferson University, Philadelphia, PA, USA; ^2^Department of Pediatrics, Nemours/Alfred I. duPont Hospital for Children, Wilmington, DE, USA

## Abstract

Acute-onset obsessive-compulsive disorder can be challenging, especially when triggered by an underlying disease process. Clinicians often turn to Pediatric Autoimmune Neuropsychiatric Disorders Associated with Streptococcal Infections (PANDAS), but it is important to consider a broad differential in these patients. We present a case of a 9-year-old girl with acute-onset obsessive-compulsive behavior likely triggered by a post-infectious phenomenon that ultimately resolved following treatment with plasmapheresis.

## 1. Introduction

Obsessive-compulsive disorder (OCD) is a debilitating disease characterized by repetitive, ritualistic, and distressing thoughts and behavior which can be very difficult to control. It affects about 1-3% of school-aged children and adolescents [1, 2]. The cause of obsessive-compulsive disorder is unknown; however, a combination of genetic and environmental factors may be contributory [3]. In a subset of patients, underlying disease processes such as infectious, postinfectious, and autoimmune phenomena can trigger OCD [4]. We report a 9-year-old girl with no significant past medical history who presented to the emergency department (ED) with acute-onset obsessive-compulsive behavior in the setting of positive anti-glutamic acid decarboxylase 65 (anti-GAD 65) and *Mycoplasma pneumoniae* antibodies. Here, we discuss her symptoms, diagnostic evaluation, and the treatment she received.

## 2. Case Presentation

Our patient was a 9-year-old previously healthy girl who presented to the ED with a chief complaint of acute-onset obsessive-compulsive behavior. Eleven days prior to presentation, she became emotionally labile, manifested as sudden episodes of sadness, withdrawal, and unwillingness to participate in social events. Five days prior to admission, her parents observed her displaying ritualistic behaviors such as watching a YouTube commercial 75 times, walking in certain patterns, taking her pajamas on and off a certain amount of times before going to school, picking her lips, and banging her ankles and the side of her abdomen against the kitchen counter. These behaviors would occupy the patient for 4-6 hours, affecting her ability to function normally. She explained that if she did not engage in these behaviors, she would continue to think about them and was worried that something bad may happen to her or her parents. She was also described as having decreased concentration and difficulty performing simple mathematical equations. This was very unusual, as her parents described her as a highly spirited and functional child who is popular at school—participating in advanced math classes, piano, tennis, and gymnastics.

Concerned with her behavior, she was taken to her primary doctor whose workup included Epstein-Barr virus (EBV) serology, anti-deoxyribonuclease (anti-DNase), antistreptolysin O (ASO), thyroid function tests (TFTs), complete blood count (CBC), comprehensive metabolic panel (CMP), thyroid-stimulating hormone/thyroxine (TSH/T4), and Lyme serology (all within normal limits). An impression of PANDAS was made, and she went home on amoxicillin and fluoxetine, which she took for two days prior to her presentation to our ED.

The family considered stress as a possible contributing factor and spent a weekend away from home for a change in environment. In their hotel room, she would ritualize walking back and forth several times. If she failed to complete these rituals, it would result in emotional outbursts and a strong urge to repeat them. Her ability to eat and drink was also impaired, causing her to become dehydrated and prompting her parents to bring her to the ED. This process took five hours due to her extreme reluctance to leave the hotel room before completing her rituals.

Review of systems was significant for the following: constitutional—negative for malaise and weight loss; skin—negative for rash, positive for bruising on ankles and waist; gastrointestinal—negative for vomiting and abdominal pain; neurologic—negative for weakness, neck pain, neck stiffness, paresthesia, and seizures.

Family history was significant for anxiety in both parents. Socially, she was living with her parents with strong support from her grandparents who live nearby. No report of sexual abuse, physical abuse, or bullying at school. Her medications included fluoxetine and amoxicillin that were started two days prior to hospitalization by her pediatrician. Allergies included oseltamivir.

On examination, she appeared well looking, in no apparent distress. Her vitals were normal; central nervous system (CNS)—neck supple, pupils equal and round, reactive to light (PEARRL), cranial nerves II-XII intact, motor, sensation, and power normal; Her affect was normal, and her mood was congruent; Head, eyes, ears, nose, and throat (HEENT)—mucous membranes dry; skin—healing bruises on her ankles and waist; cardiovascular, respiratory, and abdominal exams were normal.

In the ED, she received maintenance intravenous fluids (IVF) and was admitted to the general pediatrics floor with a presumptive diagnosis of Pediatric Acute-onset Neuropsychiatric Syndrome (PANS).

She continued to perform rituals in the hospital, requesting the participation of her parents to suppress them. When moving objects in the room, they would need to carry them over her in a certain way several times to complete the ritual without upsetting her. As her admission progressed, her urges became overwhelming, and she ultimately returned to performing the rituals herself. She paced several times across the hospital, resisting the urge to urinate until she had completed the task. This resulted in episodes of pain and distress attributed to acute urinary retention that resolved with voiding. She also regressed developmentally, showing more interest in cartoons targeted for younger children, and continued to refuse to drink, requiring IVF intermittently.

### 2.1. Diagnostic Evaluation

Psychiatry, psychology, and neurology services were consulted to help guide the patient's evaluation and treatment. She underwent a series of tests to identify a secondary cause of her presentation. This included a CBC [mild microcytic anemia], CMP [normal], Lyme serology [normal], brain magnetic resonance imaging (MRI) [mild prominence of sulci likely secondary to dehydration], repeat brain MRI about 3 weeks from the first [normal], lumbar puncture (LP) with opening pressure [normal], cerebrospinal fluid (CSF) microscopy and chemistry [normal], CSF autoimmune profile [normal], electroencephalogram (EEG) [normal], serum autoimmune encephalopathy panel [positive for anti-GAD 65 at 0.05, resulted day 16], serum N-methyl D-aspartate (NMDA) antibodies [negative], and *M. pneumoniae* serum antibodies [IgG and IgM positive, resulted day 3] with confirmatory *M. pneumoniae* IgM indirect immunofluorescence assay (IFA) [positive, resulted day 3].

### 2.2. Therapeutic Interventions

Amongst the consulting services, there were varying opinions regarding the diagnosis and treatment of the patient. Psychiatry cited case reports and experiences with patients with *M. pneumoniae* infections associated with OCD that responded to azithromycin. At their recommendation, azithromycin was initiated for a 30-day course. However, this was discontinued on day 13 because she developed diarrhea in the setting of underlying dehydration, in addition to a lack of clinical improvement. At this time, psychotropic medications were not started so as not to confound the possible response to azithromycin.

Psychology attempted a reward system to encourage oral (PO) intake, but she continued to intermittently require IVF due to inadequate intake as a result of interference from her rituals.

On day 9 of hospitalization, psychiatry recommended starting intravenous methylprednisolone (1.5 mg/kg/dose), however, no clinical response was observed after a 5-day course. Lorazepam was initiated as needed for agitation and ritual control at this time.

Due to the lack of response to the above treatments (azithromycin, reward system, steroids), intravenous immunoglobulin (IVIG) was considered, however not initiated due to a national shortage. The ultimate decision to attempt plasmapheresis was made on day 16 of hospitalization when the anti-GAD 65 was found to be positive. After her first session of plasmapheresis, she showed some improvement— she was able to participate in Halloween activities (trick-or-treat), and her rituals were less repetitive with an improvement in her PO intake. She received a total of five rounds of plasmapheresis before her compulsions decreased significantly. Sertraline 10 mg daily was initiated on day 23 of hospitalization. She was ultimately discharged after about one month of inpatient care, and continued to follow up with her outpatient psychologist, psychiatrist, and pediatrician. Her outpatient psychiatrist increased her sertraline dosage to 15 mg and added Vitamin D 1000-2000 units daily. She also completed an outpatient intensive behavioral health program after her discharge. Within a few weeks of her discharge, her compulsions and intrusive thoughts had dissipated completely.

## 3. Discussion

Our patient was positive for *Mycoplasma pneumoniae* IgM and IgG in the setting of a recent upper respiratory infection (URI), suggesting an antecedent *M. pneumoniae* infection. The medical literature describes case reports of the association of *M. pneumoniae* with acute-onset OCD and other neuropsychiatric disorders [5, 6]. She was also positive for anti-GAD 65 antibodies, which are reportedly associated with reduced gamma-aminobutyric acid (GABA) transmission and can lead to seizures, increased anxiety, behavioral changes, and encephalitis [7]. However, anti-GAD 65 levels > 20 have a higher positive predictive value for encephalitis, and her level of 0.05 is unlikely to be clinically significant [8].

She did not respond to azithromycin despite receiving a 13-day course. We hypothesize that she did not have an active *M. pneumoniae* infection on presentation, rendering the antibiotics ineffective, but may have experienced a post-infectious process in the form of *M. pneumoniae*-induced antineuronal antibodies.

She also received steroids with minimal effect. Steroids are estimated to reduce antibody production by 10-20 percent [9], which may not be an adequate nadir to produce a clinical response. However, the dramatic response to plasmapheresis suggests that the inciting antibodies were ultimately cleared and no longer exerting their antineuronal effect. Whether this improvement was secondary to clearance of *M. pneumoniae*-associated antibodies or anti-GAD 65 antibodies remains unclear.

Our patient's initial diagnosis was PANDAS/PANS which is a controversial diagnosis with conflicting evidence in the medical literature. The diagnostic criteria for PANDAS were first proposed in 1998 [4], and further revised as PANS in 2012 to include patients without group A streptococcal (GAS) infection [10], but are beyond the scope of this discussion.

Some authors have proposed steering away from PANDAS/PANS and coining childhood acute-onset neuropsychiatric syndrome (CANS) as an umbrella term with a strong emphasis on identifying the underlying etiology [11]. Using this approach, the differential diagnosis for any child presenting with neuropsychiatric symptoms includes infections, postinfectious sequelae, autoimmune disease, drug-induced, metabolic, traumatic, primary psychiatric disorder, and idiopathic, which we have listed as a guide to clinicians (Table 1).

A list of investigations has been proposed to guide clinicians (Table 2) [11]; however, no consensus has been documented. Clinicians should therefore obtain a thorough history and physical examination in order to formulate a comprehensive differential diagnosis, appropriate investigations, and a management plan. In patients with severe symptoms such as ours, an extensive evaluation is warranted.

Limitations of our evaluation included the lack of throat culture for GAS, which was arguably appropriate as the patient had no URI symptoms. More importantly, an ANA was not performed, which is commonly overlooked. This is an important test to consider as childhood-onset SLE can present with neuropsychiatric symptoms in two-thirds of patients [12].

For treatment, antibiotics should be used only when an active infection has been documented, for example, *M. pneumoniae*. Prolonged antibiotic use increases the risk of adverse drug reactions, as in this case, and antimicrobial resistance. The use of steroids, IVIG, and plasmapheresis should be determined on a case-by-case basis, strongly evaluating the risk-to-benefit ratio for each modality. Institutions are encouraged to develop clinical pathways and multidisciplinary team meetings to ensure appropriate use of the aforementioned modalities. Cognitive-behavioral therapy (CBT) and selective serotonin reuptake inhibitors (SSRIs) are reasonable adjuncts as the risk of harm is low.

Our patient underwent an extensive and invasive diagnostic workup, several unsuccessful treatments, and spent nearly an entire month admitted to the hospital before her symptoms improved. This is a severely debilitating condition that requires more high-quality research to fully understand its pathophysiology and evaluate the efficacy of various treatment options.

## Figures and Tables

**Table 1:** Differential diagnosis for CANS.

Infection	Metabolic	Drugs	Toxins	Autoimmune
Bacterial or viral meningitis, encephalitis, acute disseminated encephalomyelitis (ADEM)	Hypoglycemia, diabetic ketoacidosis (DKA), hyperammonemia, hyperthyroidism, hypothyroidism, uremia	Steroids, antihistamines, antipsychotics, phencyclidine (PCP), methamphetamine, benzodiazepines, anticholinergics, cannabis, cocaine	Organophosphates, heavy metals, alcohol	NMDA, *α*-amino-3-hydroxy-5-methyl-4-isoxazolepropionic acid (AMPA), GABA receptor antibodies, Hashimoto's, antiphospholipid, systemic lupus erythematosus (SLE)

Vascular	Psychiatric	Idiopathic		
Stroke, hypertensive emergency, subdural hemorrhage, epidural hemorrhage	Primary psychiatric disorder	No underlying etiology identified		

**Table 2:** List of suggested investigations for CANS.

Blood	CSF	Brain imaging
CBC, CMP, ammonia, erythrocyte sedimentation rate (ESR), C-reactive protein (CRP), ASO, anti-DNAse B, TSH, T4, anti-thyroglobulin, anti-thyroperoxidase, urine drug screen (UDS), ethanol level, acetaminophen and salicylate levels, antinuclear antibodies (ANA), autoimmune encephalitis panel	Cell count, differential, glucose, protein, gram stain, bacterial and viral polymerase chain reaction (PCR), autoimmune encephalitis panel, oligoclonal bands	Video EEG, Computed tomography (CT), MRI
